# Improved quantification of *Fusarium pseudograminearum* (Fusarium crown rot) using qPCR measurement of infection in multi-species winter cereal experiments

**DOI:** 10.3389/fpls.2023.1225283

**Published:** 2023-08-02

**Authors:** Andrew Milgate, Brad Baxter, Steven Simpfendorfer, Herdina, Daniele Giblot-Ducray, Nannan Yang, Beverly Orchard, Ben Ovenden

**Affiliations:** ^1^ NSW Department of Primary Industries, Wagga Wagga Agricultural Institute, Wagga Wagga, NSW, Australia; ^2^ NSW Department of Primary Industries, Tamworth Agricultural Institute, Tamworth, NSW, Australia; ^3^ South Australian Research and Development Institute, Plant Research Centre, Urrbrae, SA, Australia

**Keywords:** Fusarium crown rot (FCR), qPCR, wheat, barley, tolerance, partial resistance

## Abstract

Fusarium crown rot (FCR) causes significant grain yield loss in winter cereals around the world. Breeding for resistance and/or tolerance to FCR has been slow with relatively limited success. In this study, multi-species experiments were used to demonstrate an improved method to quantify FCR infection levels at plant maturity using quantitative PCR (qPCR), as well as the genotype yield retention using residual regression deviation. Using qPCR to measure FCR infection allowed a higher degree of resolution between genotypes than traditional visual stem basal browning assessments. The results were consistent across three environments with different levels of disease expression. The improved measure of FCR infection along with genotype yield retention allows for partitioning of both tolerance and partial resistance. Together these methods offer new insights into FCR partial resistance and its relative importance to tolerance in bread wheat and barley. This new approach offers a more robust, unbiased way to select for both FCR traits within breeding programs. *Key message:* Genetic gain for tolerance and partial resistance against Fusarium crown rot (FCR) in winter cereals has been impeded by laborious and variable visual measures of infection severity. This paper presents results of an improved method to quantify FCR infection that are strongly correlated to yield loss and reveal previously unrecognised partial resistance in barley and wheat varieties.

## 1 Introduction

Fusarium crown rot (FCR) is an important disease of winter cereals in many cereal growing regions around the world (Smiley and Patterson, 1996; Gargouri et al., 2011; Zhang et al., 2015; Alahmad et al., 2018; Kazan and Gardiner, 2018). Economic impacts of FCR, primarily through reduced grain yields, are estimated to cost the Australian winter cereals industry (wheat and barley) AUD$97 million per annum (Murray and Brennan, 2009). The disease is caused by several *Fusarium* species which colonise the basal vascular tissues of stems and restrict both water and nutrient translocation within the plant during grain fill (Knight and Sutherland, 2016). Of the *Fusarium* species that can be the causal agents of FCR, *F. pseudograminearum* (*Fp*) is the most common species observed in Australia, whereas *F. culmorum* is found to be associated with the disease in cooler regions (Backhouse and Burgess, 2002; Akinsanmi et al., 2004; Backhouse et al., 2004; Poole et al., 2013). However, both *Fusarium* species are known to often co-exist in the same locations.

FCR infections occur frequently in conservation cropping systems where tight rotations of cereal crops and retention of cereal residue (stubble) are practised (Simpfendorfer et al., 2019). Stubble acts as a refuge for the pathogen to survive up to three years or more. Fusarium mycelia colonise the living tissue and continue to grow on the stubble residue after senescence, which then provides the inoculum source for infecting the next cereal crop or alternative hosts, including grasses such as *Phalaris*, *Agropyron* and *Bromus* species (Purss, 1969; Summerell and Burgess, 1988; Summerell et al., 1989; Summerell et al., 1990; Burgess, 2014). Soil inoculum levels, symptom development and grain yield losses are influenced by environmental factors and the types of cereals being grown (Hollaway et al., 2013). FCR infection can occur early in seedling development right through to adult stages of growth. When moisture stress occurs during flowering and grain-filling, infected stems can senesce prematurely and appear as ‘white heads’. These white heads contain no or only a few shrivelled grains. FCR can impact several components of grain yield including kernel number per head, kernel weight, stem height and straw weight (Smiley et al., 2005). FCR infection occurs in both susceptible and partially resistant genotypes (Percy et al., 2012; Knight and Sutherland, 2015; Knight and Sutherland, 2016). FCR inoculum levels and impacts on grain yield have been demonstrated across a broad range of environmental conditions including years with rainfall below the long-term average during the grain-filling period and in relatively wet years (Hollaway et al., 2013; Buster et al., 2022). Hence, an alternative for FCR resistance breeding is to conjoin FCR partial resistance and/or tolerance with stress related physiological traits such as drought, heat or moisture stress (Kazan and Gardiner, 2018). While these studies have not given definitive explanations of the molecular basis for the associations, they suggest that wheat lines with higher levels of drought tolerance are able to initiate a stronger defence response to FCR infection possibly due to lower drought stress (Su et al., 2021).

The goal of breeding programs is to achieve high grain yield but with stable performance in the presence of disease. Breeders of winter cereals around the world have had great success in protecting yields from *Puccinia* species and *Zymoseptoria tritici* by combining major and minor resistance genes in resistance breeding programs (Raman and Milgate, 2012; Ellis et al., 2014; Brown et al., 2015). Breeding for FCR resistance currently focuses on identifying genotypes with partial resistance that reduces the development of basal stem browning symptom. Studies in segregating crosses or association panels have identified QTLs for resistance on at least 13 of the 21 wheat chromosomes (Collard et al., 2006; Ma et al., 2010; Liu and Ogbonnaya, 2015; Martin et al., 2015; Rahman et al., 2020; Rahman et al., 2021). However, these QTLs fail to be widely utilised in breeding programs because of the complex nature of inheritance, partial effectiveness, and poor agronomic performance including low yield potential (Rahman et al., 2020). Therefore, for FCR there is little effective resistance deployed, and all varieties are infected and suffer losses to some extent (Kazan and Gardiner, 2018). Tolerance, the ability to retain yield in the presence of infection, is frequently discussed in studies examining genotype effects of FCR and how it differs from partial resistance (Kazan and Gardiner, 2018; Forknall et al., 2019). Differences in tolerance are known to exist between winter cereal species. Barley is rated more tolerant than bread wheat which is more tolerant than durum wheat (Hollaway et al., 2013). However, difficulties exist to experimentally distinguish clear phenotypic differences between tolerance and partial resistance in field studies. Improved methods for selecting more resistant varieties are urgently needed because there are currently no effective available fungicide control options that consistently prevent yield losses from FCR.

Typically, detailed yield assessments at multiple levels of disease intensity are required to measure FCR tolerance accurately (Forknall et al., 2019). By exposing varieties to increasing levels of disease burden, from low to high, and measuring the yield response and disease severity, the rate of change in yield due to disease can be calculated as an estimate of tolerance (Forknall et al., 2019). However, in the case of FCR, these estimates of tolerance are compromised by the lack of a true nil disease comparison. This is because establishing field trial plots with no disease remains a challenge due to the ineffectiveness of fungicides and the likelihood of background presence of FCR in the target environments. Disease assessments are performed either by visual scoring of adult stem basal browning lesions or more recently by using qPCR to measure pathogen DNA load in seedlings or adults (Hogg et al., 2007; Knight et al., 2012; Liu et al., 2012; Knight and Sutherland, 2015; Knight and Sutherland, 2017; Knight et al., 2017; Ozdemir et al., 2020; Knight et al., 2021). The use of qPCR to measure Fusarium DNA levels has been shown to be positively associated with traditional disease severity methods (Hogg et al., 2007; Knight and Sutherland, 2015). Pathogen colonization, measured by qPCR, is dynamic over the lifecycle of the host. Therefore, the timing of sampling to estimate differences between genotypes must be consistent across experiments to accurately estimate differences. Knight and Sutherland (2015) detected differences in Fusarium biomass (DNA ng/g) at 16 weeks post sowing (post anthesis) and 22 weeks post sowing (harvest maturity) with the earlier sampling time showing larger differences between a relatively small set of genotypes. The large environmental interactions observed with traditional disease assessments have resulted in low heritability estimates for FCR partial resistance (Dodman and Wildermuth, 1987; Knight and Sutherland, 2015; Martin et al., 2015; Kazan and Gardiner, 2018; Rahman et al., 2020; Kelly et al., 2021). These interactions are not compatible with the selection of genotypes with combinations of multiple genes with minor effects within large breeding populations (Rahman et al., 2021).

In this study, we use multi-species experiments to estimate the genotype yield potential in treatments with (inoculated) and without (non-inoculated) FCR infection in three target environments, across two years. FCR infection was measured at maturity using quantitative PCR (qPCR), which allowed a quantitative measurement of *Fp* DNA loads to identify genotypes with partial resistance. Greater *Fp* DNA levels were strongly correlated with grain yield loss. Partitioning of tolerance and partial resistance of genotypes was achieved by using residual regression deviation as described in Kelly et al. (2021). This study is the first to measure *Fp* DNA at plant maturity in the field as a proxy for FCR infection severity and associate it with tolerance.

## 2 Materials and methods

### 2.1 Plant materials

Sixteen wheat, eight barley and one durum wheat cultivar, for a total of 25 genotypes were used to conduct these field experiments (**Table 1**). These genotypes reflected the mix of commercial cultivars grown in NSW, Australia at the time the experiments were conducted. Each genotype has a known resistance rating to FCR that ranged from moderately susceptible (MS) to very susceptible (VS) (Matthews et al., 2016). All genotypes were included in all experiments, with the exception of one barley variety, Buloke, which was present only in the Wagga Wagga 2016 field experiment.

**Table 1:** Barley, bread wheat and durum wheat genotypes included in the experiments had known crown rot resistance ratings (Crown rot rating source Matthews et al., 2016).

Species	Genotype	Crown rot rating
Barley	La Trobe	Moderately susceptible –susceptible
Barley	Commander	Moderately susceptible –susceptible
Barley	Rosalind	Moderately susceptible –susceptible
Barley	Hindmarsh	Susceptible
Barley	Compass	Susceptible
Barley	Spartacus CL	Susceptible
Barley	Bass	Susceptible
Barley	Buloke	Susceptible – very susceptible
Bread Wheat	Trojan	Moderately susceptible
Bread Wheat	Emu Rock	Moderately susceptible –susceptible
Bread Wheat	Lancer	Moderately susceptible –susceptible
Bread Wheat	Suntop	Moderately susceptible –susceptible
Bread Wheat	Merlin	Moderately susceptible –susceptible
Bread Wheat	Phantom	Moderately susceptible –susceptible
Bread Wheat	Scepter	Susceptible
Bread Wheat	Beckom	Susceptible
Bread Wheat	Corack	Susceptible
Bread Wheat	Bolac	Susceptible
Bread Wheat	DS Pascal	Susceptible
Bread Wheat	Condo	Susceptible
Bread Wheat	Flanker	Susceptible
Bread Wheat	EGA Gregory	Susceptible
Bread Wheat	DS Darwin	Susceptible
Bread Wheat	Waagan	Susceptible
Durum	Bellaroi	Very susceptible

### 2.2 Experimental design

The software package DiGGer version 1.0.5 (Coombes, 2002) in R (R Core Team, 2021) was used to generate spatially optimised randomised complete block designs for all experiments in the study. Treatments with inoculum (inoculated) and no inoculum (non-inoculated) were randomised, each with four replicates in independent experiments.

### 2.3 Field experiments

The genotypes were evaluated in three different environments, over two years. The site by year combinations and rainfall totals including annual, in-crop growing season (June to November) and during grain-filling (September or October) are outlined in **Table 2**. Field experiments were conducted at Wagga Wagga Agricultural Institute at Wagga Wagga (S -35.04419222, E 147.3167896), NSW, Australia in 2016 and 2017 and the Condobolin Agricultural Research and Advisory Station at Condobolin (S -33.064939, E 147.230877), NSW, Australia in 2017. The two previous crops at Wagga Wagga for both the 2016 and 2017 trials were lupins then canola. At Condobolin in 2017 the two previous crops were lucerne then fallow. FCR levels were not measured prior to planting. Experiments were managed with standard agronomic practices for the region. Briefly, monoammonium phosphate fertilizer (MAP) was applied at sowing (100 kg/ha at Wagga Wagga, 70 kg/ha at Condobolin) and pre-emergent herbicides were applied two days prior to sowing. Post-emergent herbicide was applied at Wagga Wagga in 2017 60 days after sowing. Foliar diseases were prevented from impacting on grain yield by targeted in-crop fungicide applications at key growth stages during September and October. Insecticide applied for aphid control in July at Wagga Wagga in 2016 and 2017. Seed rates at sowing were calculated using 1,000 grain weight and germination percentage to target the optimal number of plants per metre square (m^2^) for each site based on regional commercial best practice. Plots consisted of 6 rows of 6 m length with 24 cm spacings, average plot width was 1.44 m. After sowing plots were trimmed to 5 m length, which was the actual plot length measured at maturity for the purpose of calculating yield. Grain was harvested with a Wintersteiger plot header and yield measured as the total grain collected from the plot converted from kg/m^2^ to t/ha.

**Table 2:** Rainfall statistics for each experimental site.

Location	Year	Annual rainfall	Rainfall Decile (1-10)	GS rainfall (June-Nov)	Grain fill rainfall	Mean Grain fill rainfall	Mean GS rainfall
Wagga Wagga	2016	778.8	9	496	64	56	308
Wagga Wagga	2017	445.3	2	215	65	56	308
Condobolin	2017	490.6	6	104	6	32	180

Mean rainfall and rainfall decile figures calculated separately for each site from weather statistics available at the CSIRO Bureau of Meteorology; Wagga Wagga AMO 1941-2021 and Condobolin Agricultural Research Station 1954-2022.

Growing season (GS) rainfall June-November Wagga Wagga, June-October Condobolin, is from a sowing to maturity in each trial. Grain fill is during October at Wagga Wagga and September for Condobolin.

Sowing date was 2-4 weeks after the optimal sowing window for each experimental site. This coincided with the first week of June sowing date compared to the commercial best practice window of late April to early May. This increased the probability and extent of moisture and heat stress during grain filling, to exacerbate the expression of FCR. The experimental plot inoculation was carried out at sowing, according to the grain inoculum method described by Forknall et al. (2019) and Kadkol et al. (2021). Briefly, two grams of FCR inoculum per metre of trial row was homogenised with the viable seed in packets and sown with a plot seeder. Isolates used to create inoculum were a mixture of five *F. pseudograminearum* isolates (WAI1183, WAI1205, WAI1208, WAI1225, WAI1231) collected from southern NSW in 2013. Viable seed only was sown in the non-inoculated treatment.

*Fp* DNA was measured at harvest in all replicates of both the inoculated and non-inoculated treatments in each of the field experiments using qPCR assays delivered by the SARDI PREDICTA^®^ testing service. The sampling method ensured that the residual stubble on the outside rows and ends of the plots were avoided. All four replicates of each treatment were sampled separately with 32 stems collected from each and analysed as replicates in the qPCR analysis. The procedure to sample 32 stems per plot was as follows: each plot consisted of six rows, the inner four rows were sampled to prevent plot edge effects. Each row was 5 m long, sampling was carried out on the inner 4 m to avoid plot end effects. The 4 m sections sampled were divided into 50 cm lengths and a stem chosen at random within the 50 cm, giving eight stems per row and 32 stems per plot. The stem was trimmed to a uniform length of 5 cm and leaf sheath removed, ensuring the crown and first node was present with 32 random pieces per plot homogenised into 500 grams of pre-sterilised soil prior to analysis. The soil was sourced from the Wagga Wagga Agriculture Institute then sterilised twice on alternate days to remove any background levels of *Fusarium* species in an autoclave at 121 °C and 115 kpa for 60 minutes.

### 2.4 Fp DNA quantification

The 500g soil samples containing stem pieces were sent to the South Australian Research and Development Institute (SARDI) for DNA extraction and qPCR analysis using the commercially available PREDICTA^®^ diagnostic service (Ophel-Keller et al., 2008). The efficiency and consistency of the SARDI method to extract DNA from soil has previously been confirmed in comparison to commercial methods (Haling et al., 2011). Prior to DNA extraction, a standard amount of internal control was added to each sample to monitor both DNA extraction efficiency and PCR inhibition. The DNA extracted from soil samples was diluted 1/5 prior to analysis. Fusarium and internal control DNA levels were quantified by qPCR.

Three TaqMan MGB assays were used to quantify Fusarium DNA levels in the samples. *Fp* test 1 and test 2 respectively detect the two genetically distinct *Fp* groups present in Australia (Bentley et al., 2008). The *Fcg* test detects both *F. culmorum* and *F. graminearum.* The *Fcg* test was included to detect any background FCR infection by the main non-target *Fusarium* spp. Primers and probes for each test were designed in the Internal transcribed spacer of ribosomal DNA, with amplicon size ranging from 64 to 130 base pairs (**Table 3**). The specificity of each test was confirmed using pure genomic DNA of their respective target and closely related species; the *Fcg* test also detect *F. cerealis* and *F. crockwellense* (not shown). A calibration standard was prepared for each test, using pure genomic DNA from their respective target species. Each test was shown linear over seven orders of magnitude using a 10-fold dilution range of the calibration standard from 200,000 fg/ul to 2 fg/ul prepared by direct dilution. The limit of detection of all the tests was less than 2fg/ul; efficiency of the tests were shown to be between 91.7 and 93.9 (**Table 3**). qPCR were performed in singleplex on QuantStudio7 Flex real-time PCR system (Applied Biosystems, Foster City, CA, USA), in 10 μL volume containing 4 μL DNA, 200nmol/L TaqMan probe and 400 nmol/L each primer in 1× Quantitect Probe PCR master mix (Qiagen, Hilden, Germany). Cycling conditions were 15min at 95°C followed by 40 cycles of 15 s at 95°C and 1 min at 60°C. Each PCR plate included no-template controls as well as calibration standards to calculate the amount of *F. p* and *F. c/g* DNA; results were reported as Log10 pgDNA per gram of sample.

**Table 3:** Characteristics of the *Fusarium pseudograminearum* (*Fp* test 1 and *Fp* test 2) and *Fusarium culmorum/graminearum* (*Fcg*) qPCR tests used to detect Fusarium DNA levels including forward (F) primer, reverse (R) primer and probe sequences, amplicon size in base pairs (bp) and test efficiency.

Test	F primer (5’-3’)	R primer	Probe	Amplicon size (bp)	Efficiency (%)
*Fp* test 1	GTTGGGAGCTGCGTCCG	CAACATTCAGAAGTTGGGGTCTA	6FAM-CACTCCCCAAATACA	130	92.1
*Fp* test 2	GTTGGGAGCTGCGTTA	CAACATTCAGAAGTTGGGGTGTT		130	91.7
*Fcg*	TGGGAGCTGCAGTCCTGCT	ACGCTATGGAAGCTCGACGT		64	93.9

The TaqMan probe used for all three tests is minor grove binding (MGB) and labelled with the FAM fluorophore.

No significant infections with *F. culmorum/graminearum* were observed with only 21 plots from the 592 tested returning positive results (15 plots less than Log_10_ 1 pgDNA/gram, 1 plot less than Log_10_ 2 pgDNA/gram and 5 plots less than Log_10_ 3 pgDNA/gram). For analysis the sum of *Fp* test 1 and test 2 were used as the surrogate for total FCR infection. Control samples of ‘sterilised soil only’ were submitted for analysis in 2016 and 2017 no *Fusarium* DNA was detected in these samples.

### 2.5 Phenotypic data analysis

Phenotype records for grain yield (t/ha) and *Fp* DNA (Log_10_ *Fp* pgDNA/gram) were modelled separately first using a univariate multiplicative mixed linear model following the approach of Gilmour et al. (1997). The univariate model used for each trait is described as follows:


$$y_{1}=X_{1}\tau_{1}+Z_{1}u_{1}+e_{1}$$

In each model $y_{1}$ is the data vector of the response variable; $\tau_{1}$ is a vector of fixed effects (including genotype, trial and treatment effects, all two and three way interaction terms of these effects and the intercept) with associated design matrix $X_{1}$ . The significance of fixed effects and interaction terms for these models is given in **Supplementary Table 1**. The term $u_{1}$ is a random component with associated design matrix $Z_{1}$ and contains the experimental blocking structures (including replicate) used to capture extraneous variation. The residual error $e_{1}$ was assumed to have distribution $e_{1}∼N(0,\sigma^{2}R$ *)* where $\sigma^{2}$ is the residual variance for the experiment and R is a matrix that contains a parameterization for a separable autoregressive AR1⊗AR1 process to model potential spatial correlation of the observations.

For the next step of the analysis, phenotype records for four traits (grain yield non-inoculated (t/ha), grain yield inoculated (t/ha), disease severity non-inoculated (Log_10_ *Fp* pgDNA/gram), disease severity inoculated (Log_10_ *Fp* pgDNA/gram) were modelled using a multivariate multiplicative mixed linear model. Records for two traits (*Fp* DNA non-inoculated, *Fp* DNA inoculated) were log transformed before modelling. The model used is described as follows:


$$y_{2}=X_{2}\tau_{2}+Z_{2}u_{2}+e_{2}$$

where $y_{2}$ is a vector of length n=4×296 containing stacked vectors for the four traits. $\tau_{2}\;$ is a vector of fixed effects including trait means for the design matrix $X_{2}.$ The term $u_{2}$ is the vector of genotype effects for each trait corresponding to the experimental design structure $Z_{2}$ . The vector $e_{2}$ of length n containing the residuals of the four traits was modelled with an unstructured variance-covariance matrix between traits. This structure permits the fitting of linear relationships at the residual level between the four traits. Modelling was performed using the R software package ASReml-R version 4.1.0 (Butler et al., 2017), in the R statistical software environment (R Core Team, 2021). Overall genotype Best Linear Unbiased Predictions (BLUPs) for the four traits across experiments were predicted from the multivariate mixed model and the BLUPs for *Fp* DNA non-inoculated and *Fp* DNA inoculated traits were back-transformed.

The linear relationships between each of the four traits was determined from the trait: genotype covariance modelling using methods detailed in Zhang et al. (2019) as follows:


$$e_{A}=\beta_{1}e_{B}+\beta_{0}$$

Where A and B refer to the two traits in each respective pairwise comparison between the four traits modelled, and where the slope of the regression is calculated as:


$$\beta_{1}=\frac{\sigma_{AB}}{\sigma^{2}{\;}_{A}}$$

and the intercept $\beta_{0}$ was determined from the overall genotype Best Linear Unbiased Predictions (BLUPs) for the four traits across experiments from the multivariate mixed model. The difference (residual) between the BLUP from the mixed model for the response trait in each pairwise comparison and the predicted value on the trend line was calculated. The deviation from the regression values for the pairwise comparison between grain yield inoculated and grain yield non-inoculated traits are referred to hereafter as retention values. The deviation from the regression values for the pairwise comparison between grain yield inoculated and *Fp* DNA inoculated traits are referred to hereafter as tolerance values.

## 3 Results

Seasonal conditions varied considerably across the three experiments, resulting in differences across sites and treatments for yield (**Tables 2**, **4**). Rainfall was above average during the growing season (GS) at Wagga Wagga in 2016. Below average GS rainfall was received at Wagga Wagga in 2017 and Condobolin in 2017, reaching only 70% and 57% of their annual mean rainfall respectively (**Table 2**). The range in moisture conditions allowed for differences in expression of tolerance and partial resistance to *Fp* DNA to occur.

**Table 4:** Summary table of mean treatment effects for grain yield (t/ha) and *Fp* DNA of crown rot infection (*Fusarium pseudograminearum* Log_10_ pgDNA/gram) at each experiment.

EXPT	Treatment	No. of Plots	*Fp* DNA (Log_10_ pgDNA/gram) ± 95% ci	p value	Grain yield (t/ha)	p value
2016 Wagga Wagga	Non-inoculated	96	0.52 ± 0.16	<0.0001	5.379 ± 0.15	0.4514391
2016 Wagga Wagga	Inoculated	96	3.40 ± 0.16		5.208 ± 0.188	
2017 Wagga Wagga	Non-inoculated	100	0.27 ± 0.08	<0.0001	3.891 ± 0.111	0.0006582
2017 Wagga Wagga	Inoculated	100	3.79 ± 0.23		3.511 ± 0.126	
2017 Condobolin	Non-inoculated	100	2.17 ± 0.30	<0.0001	1.305 ± 0.081	0.0027557
2017 Condobolin	Inoculated	100	4.51 ± 0.06		0.964 ± 0.088	

Non-inoculated = plots sown without crown rot inoculum, Inoculated = plots sown with crown rot inoculum. Inoculum was *Fusarium pseudograminearum*. Means for all experiments with 95% confidence interval (ci), p-value for paired comparison of treatments is given at the experiment level.

FCR infection occurred in all experiments, with variation occurring in levels of *Fp* DNA between genotypes and treatments (**Table 4**). Low levels of *Fp* DNA were detected in all non-inoculated treatments across the three field experiments. Significant differences ( p<0.0001 ) of *Fp* DNA (qPCR) were observed between treatments at all three sites (**Table 4**). The qPCR results discriminated the *Fp* DNA levels between genotypes in the inoculated treatments and non-inoculated treatments (**Supplementary Table 2**). The below average GS rainfall in 2017 at both Wagga Wagga and Condobolin contributed to lower grain yield overall and significant losses in the *Fp* inoculated treatment (**Table 4**). While at Wagga Wagga 2016 the above average GS rainfall contributed to higher yield and no significant loss was recorded due to *Fp* treatment despite significant differences in *Fp* DNA being measured between the inoculated and non-inoculated treatments (**Table 4**).

The univariate models for yield and *Fp* DNA reveal that the fixed effects for each trait model are significant (Wald Chi-Squared Test) at p < 0.05 for the trait mean, the experiment mean, genotype and treatment (**Supplementary Table 1**). The two-way interaction terms for the yield model (genotype × treatment, experiment mean × genotype and experiment mean × treatment) were significant at p < 0.05 . The two-way interaction terms genotype × treatment and experiment mean × treatment for the *Fp* DNA model were not significant. The three-way interaction term experiment mean x genotype × treatment was significant for the *Fp* DNA model only.

### 3.1 Yield

Grain yield was reduced by FCR infection in the three field experiments and was significant for Wagga Wagga in 2017 ( p=0.0006582 ) and Condobolin in 2017 ( p=0.0027557 ) (**Table 4**). Yield at the three sites was in accordance with the seasonal rainfall patterns (**Table 2**). The lowest site mean yield (1.305 t/ha) in the non-inoculated treatment was recorded at Condobolin in 2017 whilst the highest (5.379 t/ha) was measured at Wagga Wagga in 2016.

**Figure 1:**
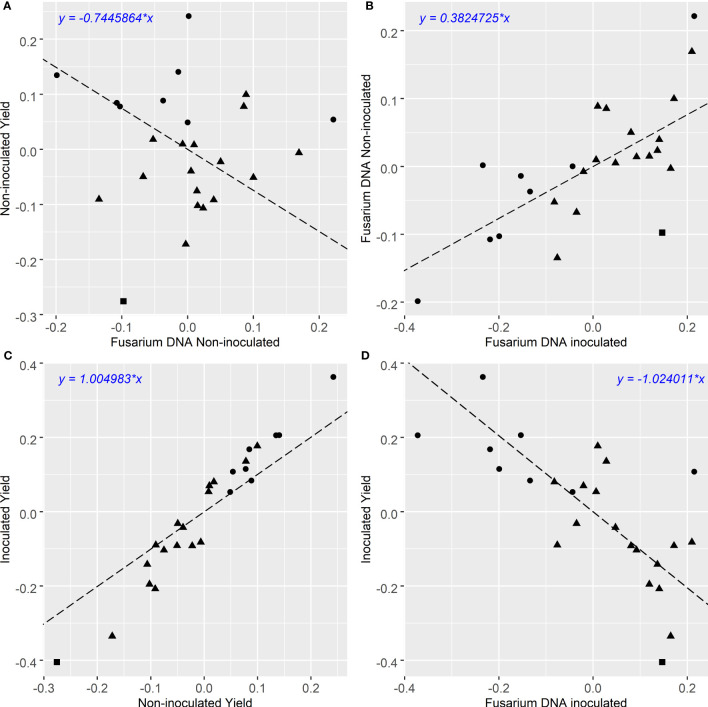
**(A–D)**: Comparison of grain yield and qPCR CR infection BLUPs of winter cereals from 3 experiments. Grain yield in t/ha, Fusarium DNA is qPCR of *F. pseudograminearum* in Log_10_ pg DNA/gram. Triangle, square and circle symbols indicate bread wheat, durum wheat and barley genotypes respectively. Pearson’s correlation coefficient for each comparison is **(A)**: *r* = 0:018 *p* < 0:9312, **(B)**: *r* = 0:715 *p* < 0:00006, **(C)**: *r* = 0:974 *p* < 0:000001, **(D)**: *r* = −0:715 *p* < 0:00006.

**Figure 2:**
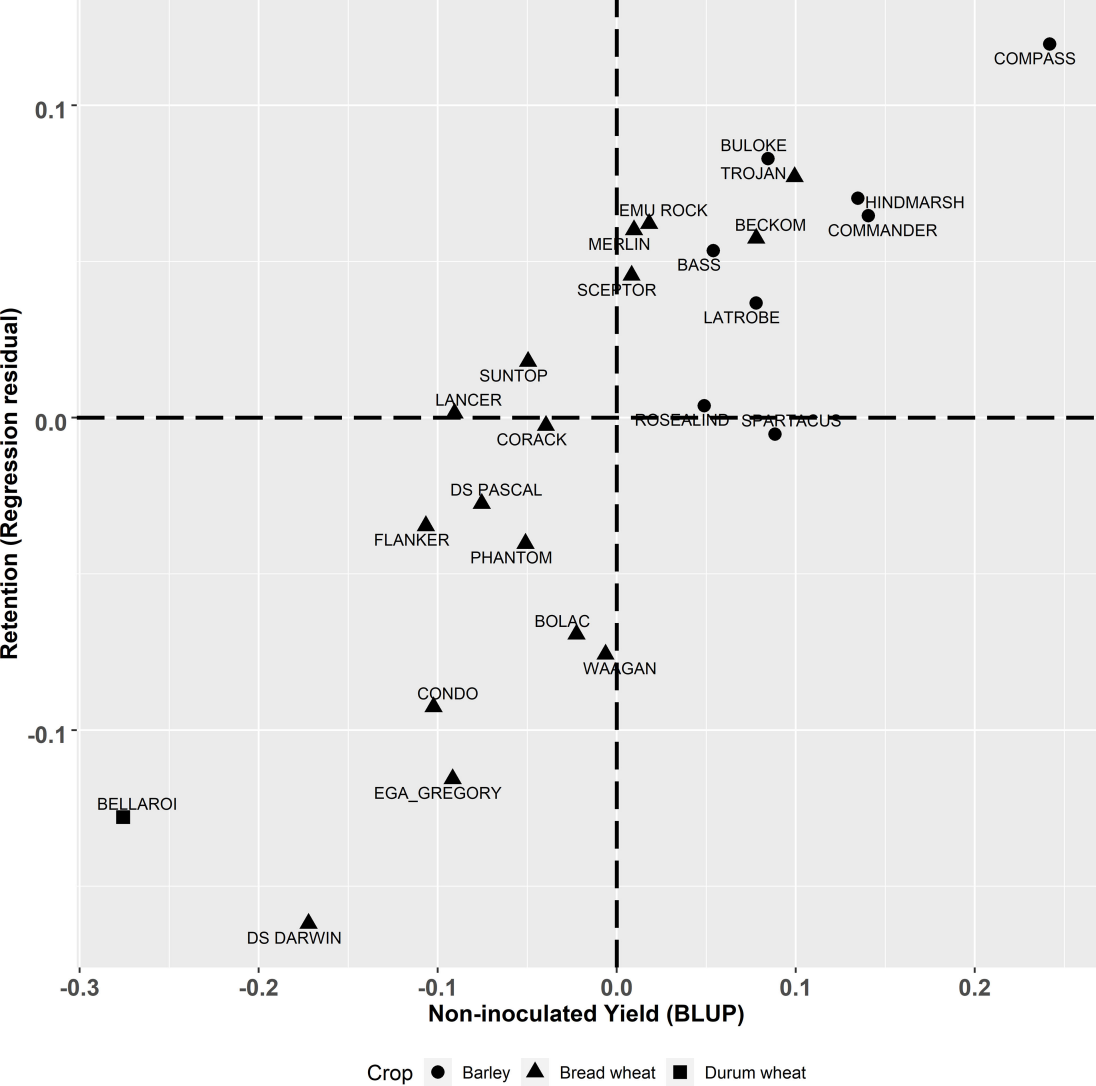
Best linear unbiased predictors (BLUPs) of genotype effects from the multivariate analysis for the derived trait of yield retention (t/ha) (regression of residuals) plotted against yield (t/ha) in non-inoculated plots. Triangle, square and circle symbols indicate bread wheat, durum wheat and barley genotypes respectively.

The multivariate analysis of yield under two contrasting levels of disease had a high correlation of r=0.974 ( p<0.000001 ) (**Figure 1C**). Barley genotypes had higher yield compared to most of the bread wheat genotypes in both the inoculated and non-inoculated treatments at each site. Within the inoculated treatment, when comparing the same cereal species, there were large genotype differences in achieved yield. When compared to the combined experimental yield mean (**Supplementary Table 2**), some wheat genotypes have yields lower than the mean bread wheat yield and ranged from -0.335 to +0.177 t/ha of the combined mean whilst mean barley yield were all higher than the experimental mean and ranged from +0.108 to +0.362 t/ha. The highest yielding barley genotype in both treatments was Compass with LRBP Trojan being the highest yielding bread wheat genotype. The durum wheat genotype EGA Bellaroi was the lowest yielding of all cereal species and genotypes. The retention value of genotypes (residual deviation from the regression between the yield under inoculated and non-inoculated conditions, **Figure 1C**) is plotted against non-inoculated yield in **Figure 2**. Genotypes in the top left quadrant of **Figure 2** have above average yield in both the inoculated and non-inoculated treatments. While those in the bottom left quadrant have above average yield in the non-inoculated treatment but lower than average yield in the inoculated treatment. Genotypes in the top right quadrant have below average yield in the non-inoculated treatment but above average yield in the inoculated treatment. While those in the bottom right have below average yield in both the inoculated and non-inoculated treatments. The magnitude of the retention value detected amongst the experimental genotypes ranged from -0.16 to +0.12 t/ha. Direct comparisons can be made between genotypes which show large differences in their ability to retain yield in the presence of FCR infection, such as Waagan and Scepter. The yield of Waagan is negatively impacted under high levels of FCR infection compared to Scepter which maintained above average yield in the *Fp* inoculated treatment (**Figure 2**).

### 3.2 Fp DNA

**Figure 3:**
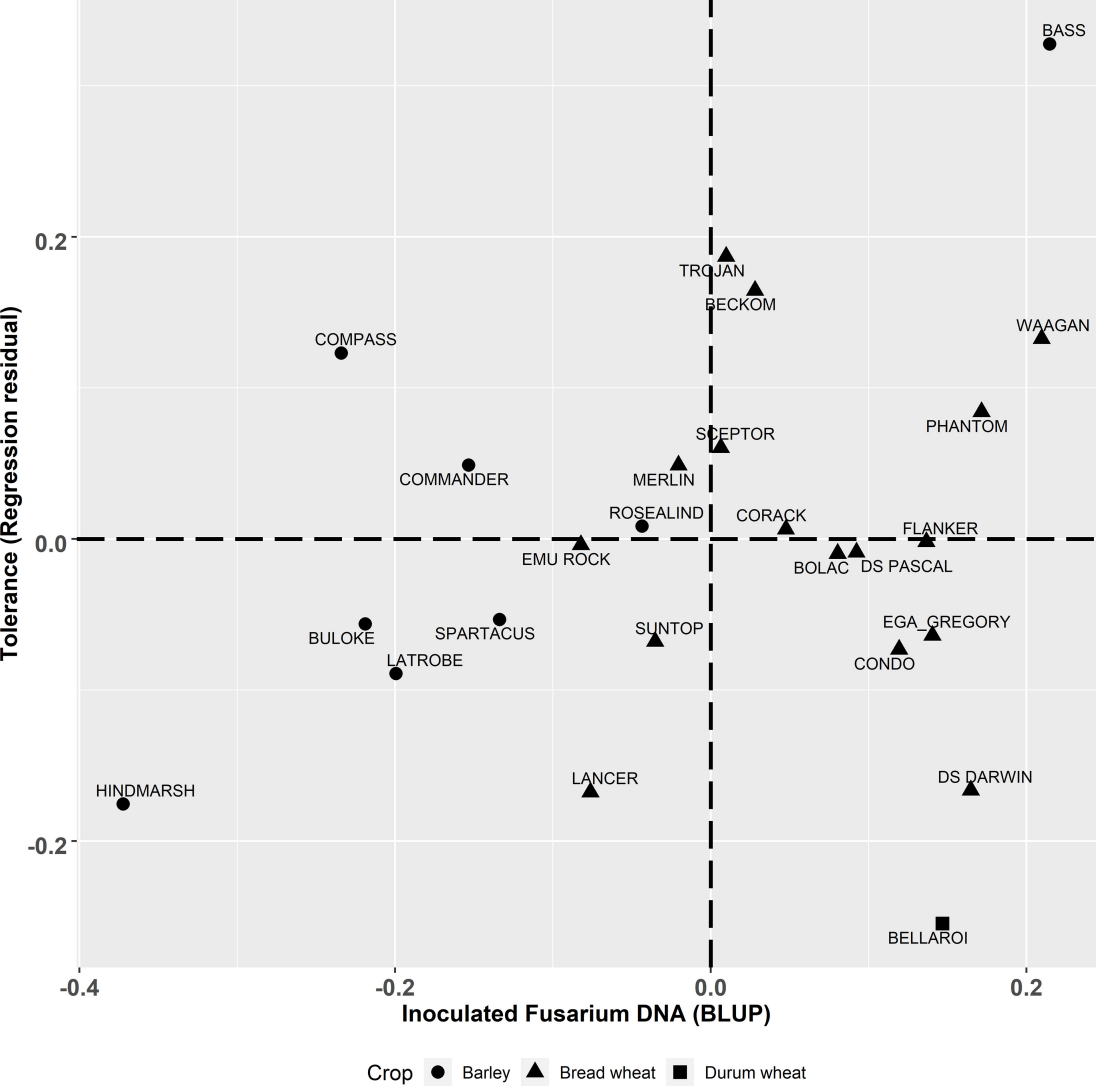
Best linear unbiased predictors (BLUPs) of genotype effects from the multivariate analysis for the derived trait of tolerance (t/ha) (regression residual) graphed against CR infection (Log_10_ pgDNA/gram) in the inoculated plots. Triangle, square and circle symbols indicate bread wheat, durum wheat and barley genotypes respectively.

Significant variations in FCR infection were detected among genotypes using Log scores of the amount of *Fp* DNA per gram of sample (Log_10_ *Fp* pgDNA/gram, **Figures 1** – **3**). Overall, the levels of *Fp* DNA were 100-1000 times higher in the inoculated treatment than in the non-inoculated treatment (**Table 4**). The individual genotype BLUPs from the 3 experiments showed the highest natural background *Fp* DNA in the non-inoculated treatment were measured in the barley genotype Bass (Log_10_ 1.215), while the highest background level detected in bread wheat was in the genotype Waagan (Log_10_ 1.163) (**Supplementary Table 2**). Both Bass (Log_10_ 4.119) and Waagan (Log_10_ 4.115) also had the highest *Fp* DNA in the inoculated treatment of all genotypes (**Figure 1B**, **Supplementary Table 2**). There was a strong positive correlation ( r=0.715, p<0.00006) between the levels of *Fp* DNA recorded for all genotypes in the two treatments (**Figure 1B**). Six out of eight barley genotypes had lower levels of *Fp* DNA than the bread wheat genotypes included in the experiments.

The degree of partial resistance (the ability of the host to lower disease levels compared to susceptible individuals) within the tested genotypes, indicated here as lower *Fp* pgDNA/gram measured by qPCR, was negatively correlated ( r=−0.715, p<0.00006 ) to yield (**Figure 1D**). However, some genotypes deviated from the regression indicating varying levels of tolerance to FCR. The tolerance value is presented (residual deviation of genotype yield performance in the *Fp* inoculated treatment) against *Fp* DNA (*Fp* pgDNA/gram) in **Figure 3**. Genotypes in the top right quadrant have above average *Fp* DNA (more susceptible) but have higher yield relative to other genotypes with the same level of *Fp* DNA, as such, showing higher tolerance to FCR. While those in the bottom right have above average *Fp* DNA (more susceptible) but lower than average yield in the inoculated treatment, displaying intolerance to FCR. Genotypes in the top left have below average *Fp* DNA (more partial resistance) in the *Fp* inoculated treatment and also above average yield (higher tolerance). While those in the bottom left have below average *Fp* DNA (more partial resistance) in the inoculated treatment but lower than average yield in the *Fp* inoculated treatment, displaying intolerance to FCR.

This study highlights the complexity of selecting for genetic improvement for FCR resistance and tolerance in winter cereals. Of the genotypes included in the study, LRBP Trojan has the highest level of resistance according to traditional phenotyping methods with a MS rating (**Table 1**). However, LRBP Trojan performed poorly, being worse than five other bread wheat genotypes when using qPCR to measure *Fp* DNA. Conversely, LRBP Trojan performed comparatively well for the tolerance measure, ranking highest of the bread wheat genotypes, illustrating it maintains yield in the presence of FCR infection through a tolerance mechanism rather than partial resistance. Using yield retention alone fails to identify the different ways cereal genotypes can achieve yield stability in the presence of FCR infection.

In another example, using qPCR to quantify *Fp* DNA reveals different responses to FCR, which can be seen in barley genotypes Hindmarsh and Commander. Both genotypes rank highly for yield retention (**Figure 2**). However, when their partial resistance and tolerance are separated (**Figure 3**), they display contrasting adaptations to FCR infection. Hindmarsh in this study demonstrated the highest level of partial resistance of all barley genotypes, but it was the second least tolerant genotype to FCR infection. Commander combines some partial resistance with some level of tolerance to achieve the highest yield retention rank of the barley genotypes examined in this study.

## 4 Discussion

This study has demonstrated in multi-cereal species experiments, an improved method to estimate the genotype yield potential in the presence of FCR infection by using the residual regression deviation as a measure of yield retention in combination with qPCR analysis of *Fp* levels at harvest as a measure of partial resistance. The use of qPCR to measure *Fp* DNA in cereal stubble at maturity, allowed a higher degree of resolution between genotypes than traditional rating by visual browning assessments of stem bases shown in **Table 1**. The three experiments experienced different environmental conditions which resulted in varying levels of *Fp* DNA and yield impact. The improved measure of *Fp* DNA allows for the partitioning and selection for both tolerance and partial resistance within winter cereal genotypes. Together these methods offer new insights into *Fp* DNA and its relative importance to tolerance in bread wheat and barley. It also provides a more robust technique to select for both traits within breeding programs compared with visual assessments which are more subjective and variable between multiple operators. The increased throughput and accuracy of qPCR measurement of FCR means this method is likely to be more useful in assessment of lower infection measured as *Fp* DNA and tolerance in cereal breeding programs, and as such is more likely to be useful as a selection tool.

qPCR quantification of *Fusarium* DNA at maturity, as an alternative measure of visual FCR severity, improved the correlation between infection and yield loss. This study is the first to measure *Fp* DNA at plant maturity under field conditions and associate it with tolerance. Previous studies using measures of *Fusarium* DNA have been conducted under controlled environment conditions in seedlings and have not been then associated to yield outcomes. Several studies have applied qPCR to inoculated seedlings which were up to 7 weeks old (Knight et al., 2012; Liu et al., 2012; Ozdemir et al., 2020). They found *Fusarium* DNA levels in seedlings were correlated to visual disease scores of seedlings for some wheat varieties, however there were inconsistencies observed where symptoms did not match the high or low levels of DNA detected. In adult plants, the correlation between visual symptoms and *Fusarium* DNA levels was shown to be impacted by the timing of collection and choice of stem sections to assess, but was improved when sampling was conducted during post-anthesis to early milk development growth stage and differences were detected between genotypes at maturity (Knight and Sutherland, 2015; Knight et al., 2017; Knight et al., 2021). In contrast to our study Liu et al. (2012) and Knight and Sutherland (2017) observed that in seedlings, barley genotypes were more susceptible to infection than the bread wheat entries used. However, two studies have found barley has lower yield loss to FCR compared to bread wheat and this attribute has been associated with higher levels of tolerance (Klein et al., 1989; Hollaway et al., 2013). This study reveals for the first time that barley varieties have higher levels of partial resistance at maturity than bread wheat, as well as higher tolerance, with both traits making contributions to increased yield performance in the presence of FCR infection.

The *Fp* DNA determined by qPCR in this study had a strong negative correlation with yield in the presence of disease. Traditional visual methods of measuring FCR severity on the basis of browning of stem bases have not been found to be strongly correlated with yield outcomes (Kelly et al., 2021; Rahman et al., 2021). The suggested reasons are the complex environmental influence on FCR symptom expression and the extent of yield loss suffered by winter cereals (Hollaway et al., 2013). Our method provides a quantitative measurement of phenotype that can be used to identify differences in partial resistance more accurately which is less subjective than previous visual measurements. Partial resistance is determined by the lower relative amounts of*Fp* DNA in plant tissue among a collection of host genotypes.

Our method allows for selection of both partial resistance and tolerance traits with greater confidence. Following the method of Kelly et al. (2021), this study has been able to effectively separate partial resistance and tolerance effects contributing to yield retention in the presence of FCR infection. Estimation of tolerance relies on a precise and repeatable measure of disease severity so that differences in yield at a given level of pathogen burden, reflect adaptations other than resistance, leading to reduced yield loss. The correlation between disease severity (stem basal browning) and yield was weaker (r= -0.19) in Kelly et al. (2021) compared to this study (*Fp* DNA) at ( r=−0.715 ). Rahman et al. (2021) also found that the visual assessment of basal browning had low heritability and a poor correlation with yield in the presence of FCR infection. In this study, the correlation between yield in different genotypes in inoculated and non-inoculated treatments was very strong ( r=0.974 ) as in Kelly et al. (2021) (r= 0.95), which is notable. These findings suggest the yield effects of FCR infection on genotypes are relatively stable across environments and it is the visible disease severity symptoms which are variable due to environmental interactions or rating method variations.

This study of multi-cereal species in multiple environments shows there is genetic potential to improve yield performance of wheat in the presence of FCR infection. The results confirm that barley genotypes have better yield performance in the presence of disease, and this is not solely due to adaptive traits such as shorter growing season providing escape or tolerance to disease expression, but also involves higher levels of partial resistance. Hollaway et al. (2013) showed barley yield loss was less than bread wheat and durum wheat yield loss under FCR disease pressure. The findings were not equated to measurement of FCR severity nor did it associate disease symptoms with yield loss. Not all barley genotypes had superior yield over bread wheat genotypes in the presence of FCR infection, suggesting this is potentially an adaptive trait. Using traditional disease assessment methods such as stem basal browning, none of the barley varieties included in this study have high levels of partial resistance (**Table 1**). The most resistant barley genotype in this study is rated as moderately susceptible to susceptible (MSS) (**Table 1**). However, measuring infection using qPCR did reveal barley genotypes with lower levels of *Fp* DNA than bread wheat genotypes.

Kelly et al. (2021) suggests that in isolation there is limited value of retention (responsiveness, or regression residual) as a breeding trait because it does not partition the tolerance response independently from partial resistance. While in theory the pursuit of partitioning these effects is desirable, the practical application of the method at the scales required by breeding programs to screen large segregating populations makes it impractical given the resources required to apply current visual disease phenotyping methods. Yield retention as a first screen for large populations, is a simpler, unbiased approach than current more labour-intensive phenotyping methods. Yield retention alone will identify genotypes that have either a combination of both traits or high levels of either partial resistance or tolerance, but ultimately for a grower high yield in the presence of FCR infection is the overriding breeding objective. For example, the contrast between the barley genotypes Commander and Hindmarsh in our study illustrate this point, both have high yield retention (**Supplementary Table 2**). However, these two genotypes achieved this in different ways. Hindmarsh achieved higher yield through having low tolerance and higher partial resistance compared to Commander, which had higher tolerance but less resistance to FCR infection (**Figure 3**). Hence, the ability to partition and quantify these two independent traits is important from a breeding perspective but could be completed in a sequential manner to make greater genetic gain per unit of breeding input. The requirement of simultaneously measuring yield performance under high disease pressure and no disease is difficult to achieve routinely. Our results show that even when FCR hosts are excluded for two years prior to planting background inoculum can persist and infect plants. Careful testing of potential trial locations will be necessary to accurately estimate non-infected yield potential of genotypes.

## 5 Conclusions

In summary, there was a strong correlation between *Fp* DNA measured using qPCR at maturity and yield of winter cereals grown in inoculated and non-inoculated treatments under field conditions, across variable rainfall conditions over two seasons. By using a multivariate analysis, we have presented a method to increase the scale and reliability of selection for genotypes that are more yield responsive in the presence of disease, which could be used to improve selection within wheat and barley breeding programs. This study using commercial genotypes demonstrated that superior lines can be selected under field conditions. A further study using a segregating population and selection under field conditions is required to validate gains that could be achieved by implementing this strategy within a breeding program. Our use of qPCR to quantify *Fp* DNA in this study had a high degree of resolution between genotypes which allowed partitioning of tolerance from partial resistance. This method offered new insights into FCR partial resistance and its relative importance to tolerance in bread wheat and barley along with a more robust, less subjective technique to potentially select for both important traits within breeding programs.

## Data availability statement

The raw data supporting the conclusions of this article will be made available by the authors, without undue reservation.

## Author contributions

AM conceived of the study and coordinated its design and execution. AM and BB conducted the experiments and collected the data from the experiments. AM, BOr and BOven analysed the data. AM wrote the draft. H and DG-D developed and validated the qPCR tests. AM, NY, BOr, BB, SS, DG-D and BOven reviewed and wrote the manuscript. All authors read and approved the final manuscript.
