# Supplementary material for: Improved quantification of Fusarium pseudograminearum (Fusarium crown rot) using qPCR measurement of infection in multi-species winter cereal experiments
Source: Front Plant Sci. 2023 Aug 2;14:1225283. doi: 10.3389/fpls.2023.1225283 (PMC10433387; doi:10.3389/fpls.2023.1225283)
Supplement: Supplementary file 1 [file Table_1.docx]

Supplementary Table 1. Table of effects and significance for Yield and Log_10_ *Fp* DNA (pgDNA/gram). A univariate linear mixed model was fitted to each trait separately using Asreml-R (Butler et al., 2017). The model included Experiment, Genotype, Treatment and all 2-way and 3-way interactions of these terms as fixed effects. For the trait Yield random effects included replicate effects at each site and at the residual level, an autoregressive AR1 (Range) x AR1 (Row) correlation was fitted to Wagga Wagga 2016 data, Genotype (Range) x AR1 (Row) was fitted to Wagga Wagga 2017 date and Genotype (Range) x Genotype (Row) to Condobolin 2017 data. For the trait *Fp* DNA random effects included replicate effects at each site and residual variance was modelled at the Experiment x Treatment level to better reflect the variation in the data.

|  | **Yield** |  | **Log_10_ *Fp* DNA** |  |
| --- | --- | --- | --- | --- |
| **Fixed Effect** | **Wald Statistic – approx. F test** | **Probability** | **Wald Statistic – approx. F test** | **Probability** |
| Mean | F(1,26.4)=7828.0 | p<0.001 | F(1,17.1)=8187.0 | p<0.001 |
| Experiment | F(2,5.0)=404.5 | p<0.001 | F(2,9.2)=125.8 | p<0.001 |
| Genotype | F(24,272.3)=95.6 | p<0.001 | F(24,207.8)=1.6 | P=0.0435 |
| Treatment | F(1,272.3)=348.5 | p<0.001 | F(1,311.9)=1823.0 | p<0.001 |
| Genotype x Treatment | F(24,280.0)=3.2 | p<0.001 | F(24,309.1)=1.4 | P=0.1110 |
| Experiment x Genotype | F(47,276.9)=10.3 | p<0.001 | F(47,310.3)=0.9 | P=0.7054 |
| Experiment x Treatment | F(2,217.1)=4.7 | P=0.0105 | F(2,188.6)=20.8 | p<0.001 |
| Experiment x Genotype x Treatment | F(47,286.1)=1.2 | p=0.2176 | F(47,243.8)=1.5 | p=0.0221 |
